# *Allium saralicum M. Fritsch* extract improves cognitive function in male rats with streptozotocin-induced diabetes cognitive impairment

**DOI:** 10.22038/ajp.2025.26350

**Published:** 2026

**Authors:** Shima Mohammadi, Leila Karimi-Zandi, Parviz Dousti Kataj

**Affiliations:** 1 *Department of Neurosciences, School of Medicine, Shahroud University of Medical Sciences, Shahroud, Iran*; 2 *Neuroscience Research Center, School of Medicine, Shahroud University of Medical Sciences, Shahroud, Iran*; 3 *Department of Neurosciences, School of Advanced Technologies in Medicine, Golestan University of Medical Sciences, Gorgan, Iran*; 4 *Neuroscience Research Center,* *Biomedical Research Institute, Golestan University of Medical Sciences, Gorgan, Iran*; 5 *School of Cognitive Sciences, Institute for Research in Fundamental Sciences (IPMA, Tehran 1956836484, Iran*

**Keywords:** Allium saralicum M. Fritsch, Cognitive dysfunction, IGF-1, NF-κB, Streptozotocin, Neuroprotection

## Abstract

**Objective:**

The chronic metabolic disease diabetes mellitus (DM) dramatically increases the risk of mental illness and cognitive decline. *Allium saralicum M. Fritsch* (ASRMF) has demonstrated antioxidant, anti-inflammatory, and neuroprotective properties. This study aimed to investigate the effects of ASRMF on memory impairment, Insulin-like growth factor 1(IGF-1) expression, and inflammation in a streptozotocin (STZ)-induced model of cognitive dysfunction.

**Materials and Methods:**

Male Wistar rats were randomly divided into five groups (n = 7): Control, Sham, STZ, ASRMF extract, and STZ+ASRMF. Memory impairment and hypoglycemia were induced following a single intraperitoneal injection of STZ (60 mg/kg). Twenty-eight days later, animals in the treated groups received oral administration of ASRMF extract (250 mg/kg) daily for 15 consecutive days. Hyperglycemia confirmation occurred through blood glucose measurements on days 3 and 28 post-inductions, and at the end of the experiment in all groups. Spatial learning and memory performance was evaluated using the Morris water maze (MWM). Brain tissue was fixed in formalin and analyzed via immunohistochemical staining to assess IGF-1 and NF-κB levels.

**Results:**

Our results demonstrated that ASRMF extract treatment significantly improved memory performance, which correlated with increased IGF-1 expression and reduced NF-κB in the hippocampus and blood glucose levels. These findings suggest that ASRMF exerts a neuroprotective effect in the diabetic rat model, likely through its anti-inflammatory properties.

**Conclusion:**

This study underscores the therapeutic potential of ASRMF in alleviating cognitive impairment associated with diabetes mellitus. However, further investigations are warranted to elucidate the precise mechanisms underlying its neuroprotective effects.

## Introduction

Diabetes mellitus (DM) significantly reduces individuals' quality of life (Wang et al. 2018). Evidence suggests that DM affects multiple organs including the brain, leading to complications such as memory loss and cognitive dysfunction (Sebastian et al. 2023). Although the precise mechanisms remain unclear, persistent hyperglycemia is known to damage the hippocampus, a critical brain region involved in learning and memory (Gupta et al. 2023). The hippocampus is particularly vulnerable to elevated blood glucose levels, which contribute to neuroinflammation, oxidative stress, and vascular dysfunction (Dash et al. 2025).

Diabetic animal models exhibiting cognitive decline often show increased levels of pro-inflammatory cytokines (Piatkowska-Chmiel et al. 2021). This correlation is strongly supported by evidence linking elevated pro-inflammatory cytokines to neuronal damage and cognitive impairment (Chai et al. 2023; Langjahr et al. 2018; Meyer-Arndt et al. 2023).

Insulin-like growth factor-1 (IGF-1) is a potent growth factor. It plays a crucial role in neural development, neurogenesis, and inflammation modulation (Arroba et al. 2018). IGF-1 promotes the synthesis of neurotrophic factors such as brain-derived neurotrophic factor (BDNF), which are essential for normal brain development (Jeon and Ha 2015). Additionally, IGF-1 reduces the production of pro-inflammatory cytokines and inhibits microglial activation, suggesting its potential role in preventing neurodegeneration (Aberg et al. 2000; Brabazon et al. 2018).

The rising interest in herbal medicine has led to the exploration of natural compounds with neuroprotective effects (Akram et al. 2023). *Allium saralicum* M. Fritsch (ASRMF), a member of the onion family, has been traditionally used to treat gastrointestinal and hepatic disorders (Sherkatolabbasieh et al. 2017). ASRMF exhibits multiple medicinal propertiesincluding anti-inflammatory, anti-hyperlipidemic, anti-anemic, and hepatoprotective effects (Goorani et al. 2019; Moradi et al. 2019; Zangeneh et al. 2018). ASRMF is also rich in flavonoids which reduce oxidative stress and mitigate metabolic and tissue disorders associated with diabetes (Fritsch and Keusgen 2006).

The potential role of ASRMF in managing type 2 diabetes has been explored (Fazelipour et al. 2021; Mohammadi et al. 2023a). Despite these promising attributes, its effects on cognitive function in diabetic models remain unexplored. 

This study aims to evaluate the potential of ASRMF in alleviating STZ-induced cognitive impairment by assessing its impact on hippocampal IGF-1 and NF-κB expression. Spatial learning and memory performance was assessed using the Morris Water Maze (MWM). 

## Materials and Methods

### Materials

Streptozotocin powder was purchased from Sigma-Aldrich (Cat# S0130). Primary antibodies used for immunohistochemistry included rabbit anti-IGF-1 polyclonal antibody (Abcam, Cat# ab9572, RRID: AB_308724) and rabbit anti-NF-κB polyclonal antibody (Sigma-Aldrich, Cat# SAB4502615, RRID: AB_10746708).

### Animals

Six-week-old male Wistar rats (RRID: RGD_13508588), weighing 180–220 g, were obtained from the Royan Institute (Tehran, Iran). They were housed under standard laboratory conditions (22 ± 2°C, 12-hour light/dark cycle) with ad libitum access to food and water. 

### Experimental protocol

Animals were randomly divided into five groups (n = 7): Control, Sham, STZ, ASRMF extract, and STZ+ASRMF.

**Figure 1:**
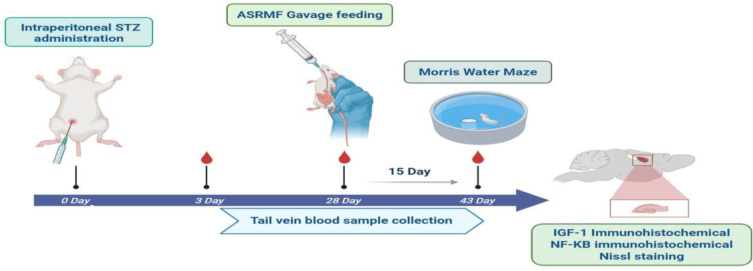
Experimental plan. Rats of the STZ group received one dose of streptozotocin intraperitoneally. After 28 days and the induction of diabetes in the rats, the animals received a hydro alcoholic-aqueous extract of ASRMF extract by gavage for 15 days. Hyperglycemia confirmation occurred through blood glucose measurements on days 3 and 28 post-induction, and at the end of the experiment in all groups. In a behavioral experiment, and the Morris water maze test was done 15 days after the ASRMF extract treatment. After sacrificing the animals, the levels of IGF-1 and NF-κB markers were measured through immunohistochemically analysis in the hippocampus tissue samples.

Hyperglycemia was induced in STZ and STZ+ASRMF groups by a single intraperitoneal injection of STZ (60 mg/kg) dissolved in citrate buffer (pH 4.5) (Ghanbari et al. 2016). After 28 days, animals in STZ+ASRMF and ASRMF extract were administered ASRMF extract 250 mg/kg/daily orally via gavage for 15 consecutive days (Ogando et al. 2021). Animals in Sham groups STZ solvent. Hyperglycemia confirmation occurred through blood glucose measurements on days 3 and 28 post-inductions, and at the end of the experiment in all groups. To assess cognitive function, the Morris Water Maze (MWM) test was performed five days prior to sacrifice. Prior to their sacrifice, rats were given medication to anesthetized using xylazine (10 mg/kg) and ketamine (70 mg/kg) (Pennasilico et al. 2025), and their brain tissues (n = 7) were collected for histological and immunohistochemical analysis (Figure 1).

### Blood glucose levels measurement

At the start of the study, all rats were screened to confirm normal blood glucose levels, and only normoglycemic animals were included. Blood glucose measurements were taken on days 3, 28, and 43 via caudal vein sampling. Three days after STZ injection, rats with blood glucose levels exceeding 120 mg/dL (Kiss et al. 2009; Santos et al. 2015), as determined by tail puncture, were classified as diabetic. Blood glucose levels were subsequently reassessed on day 28 and again at the conclusion of the experiment. The initial number of animals in each group was 14, which were then randomly assigned to the ASRMF extract and STZ+ASRMF subgroups.

### Preparation of hydro-alcoholic extract of ASRMF

The aerial parts of *Allium saralicum M. Fritsch* were collected from Kermanshah, Iran, and a voucher specimen (No. 2738RUH) was deposited at Razi University’s herbarium. The plant material was dried, powdered, and soaked in 96% ethanol for 24 hr. The resulting extract was filtered and evaporated to dryness. Previous GC/MS analysis identified key bioactive compounds, including linoleic acid, hexadecanoic acid, carvacrol, phytol, and vitamin E (Karimi Zandi et al. 2018).

### Morris water maze test

The MWM test was conducted to evaluate spatial learning and memory. A circular pool (150 cm in diameter and 60 cm deep) was divided into four quadrants. A hidden platform was placed in one quadrant, and rats were trained for four days (four trials/day). On the fifth day, a probe test was conducted to assess memory retention. Escape latency, travel distance, and time spent in the target quadrant were recorded using Ethovision software (RRID: SCR_000441)(Karimi-Zandi et al. 2022).

### Immunohistochemistry staining

Brain tissues (n=7) were fixed in 4% paraformaldehyde and sectioned (thickness of 7 µm) for immunohistochemical staining (Marcos et al., 1996). Sections were incubated with primary antibodies against IGF-1 (1:100) and NF-κB (1:50), followed by secondary antibody staining. Diaminobenzidine (DAB) was used for visualization (Mohammadi et al. 2023b). Three sections per rat were analyzed using ImageJ software (RRID: SCR_003070) and GraphPad Prism software (RRID: SCR_002798) at 200x and 400x magnification.

### Cresyl violet (Nissl) staining

To evaluate neuronal damage, brain tissues were fixed and sectioned into 7 μm slices using a rotary microtome. Following a rehydration process, sections were stained with a 0.1% Cresyl violet solution, then, rinsed in 70% ethanol, dehydrated, cleared with xylene, and mounted with coverslips (Aldana Marcos et al. 1996). The number of dark neurons as the dead cells were quantified in the 1 mm length of the CA1with using ImageJ software.

### Statistical analysis 

GraphPad Prism software was used for statistical analysis. The normality of data distribution was evaluated using the Kolmogorov-Smirnov test. Data from the Morris water maze (MWM) task, including escape latency, travel distance, and swimming speed, were analyzed using two-way ANOVA with repeated measures. One-way ANOVA followed by Tukey's post-hoc test was employed for the MWM probe trials and histological data. Results are expressed as mean ± SEM, with statistical significance at p < 0.05.

## Results

### Comparing blood sugar levels among experimental groups

**Figure 2:**
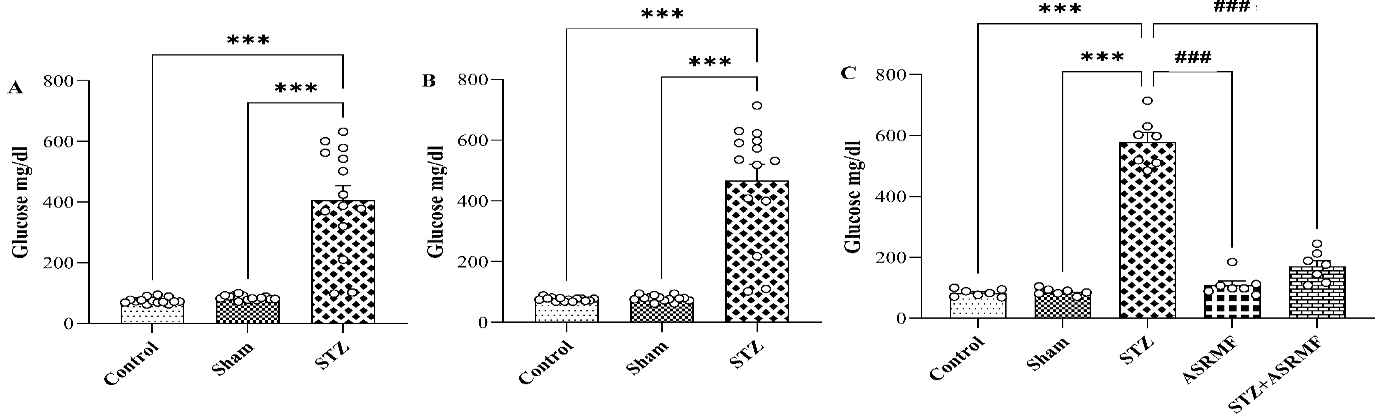
Comparison of blood glucose levels in different groups. Representative blood glucose levels are shown for (A) day 3, (B) day 28, and (C) the final day across the experimental groups. The STZ group exhibited a significant increase in blood glucose levels, indicating the induction of hyperglycemia. Treatment with ASRMF extract in the STZ+ASRMF extract group attenuated this increase. ***p<0.001 vs. control or sham and ###p<0.001 vs. STZ. Data are presented as mean ± SEM (n=14 on day 3 and 28, n=7 on the final day).

Three days after STZ injection, the STZ group showed a significant increase in glucose levels compared to the control, sham (F (2, 39) = 47.7, p < 0.001, Figure 2A), confirming hyperglycemia. This high sugar level continued on day 28 (F (2, 39) = 55.6, p<0.001, Figure 2B). The STZ+ASRMF extract group showed a significant decrease on day 28 compared to the STZ group (F (4, 30) = 149, p<0.001, Figure 2C), indicating the antidiabetic effect of ASRMF. No significant changes were seen in control, sham and ASRMF extract groups, suggesting that ASRMF extract alone does not alter normal glucose levels.

### Spatial memory in the Morris water maze 

**Figure 3:**
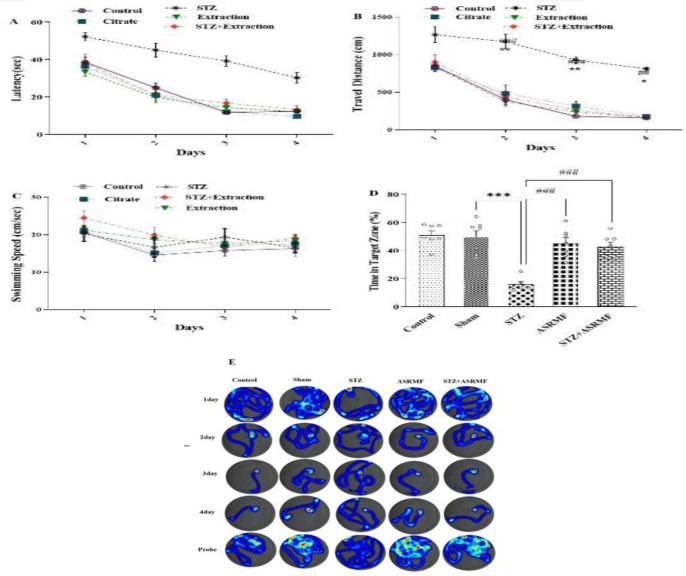
Alterations in spatial memory indices. This figure presents various spatial memory parameters assessed in the Morris water maze test. The figure shows the escape latency (a), traveled distance (b), swimming speed (c), time spent in the target quadrant (d), and a representative heat map of the traveled distance (e) for each experimental group. The control group received no drug treatment, while the sham group and STZ group were administered intraperitoneally injections of solvent and streptozotocin, respectively, to induce diabetes. Statistical analysis revealed significant differences, with the STZ group showing impaired performance compared to the sham group (*p<0.05, **p<0.01, and ***p<0.001), and the ASRMF extract treatment showing improvements in the STZ+ ASRMF extract group (###p<0.001). The data are presented as mean ± SEM, with n=7 animals per group*.*

Repeated-measures analysis revealed a significant interaction between STZ treatment and time regarding escape latency (F (12, 84) = 2.13, p=0.023, Figure 3A). Two-way ANOVA demonstrated significant effects of time (F (3, 21) = 80.9, p<0.001) and treatment group (F (4, 28) = 24.7, p<0.001, Figure 3A). No significant difference was observed between the sham and control groups in escape latency. 

A significant interaction was also found between STZ treatment and time for the distance swam to locate the hidden platform (F (12, 84) = 1.87, p=0.04, Figure 3B). Rats in the STZ group swam significantly longer distances than those in the control group (F (3, 21) = 51.3, p<0.001, Figure 3B). However, there was no significant difference in swimming speed between groups (F (4, 28) = 1.83, p = 0.15 Figure 3C). Rats in the STZ group also spent significantly less time in the target quadrant compared to the sham group (F (4, 29) = 15.9, p<0.001, Figure 3D).

### Hippocampal IGF-1 distribution 

**Figure 4:**
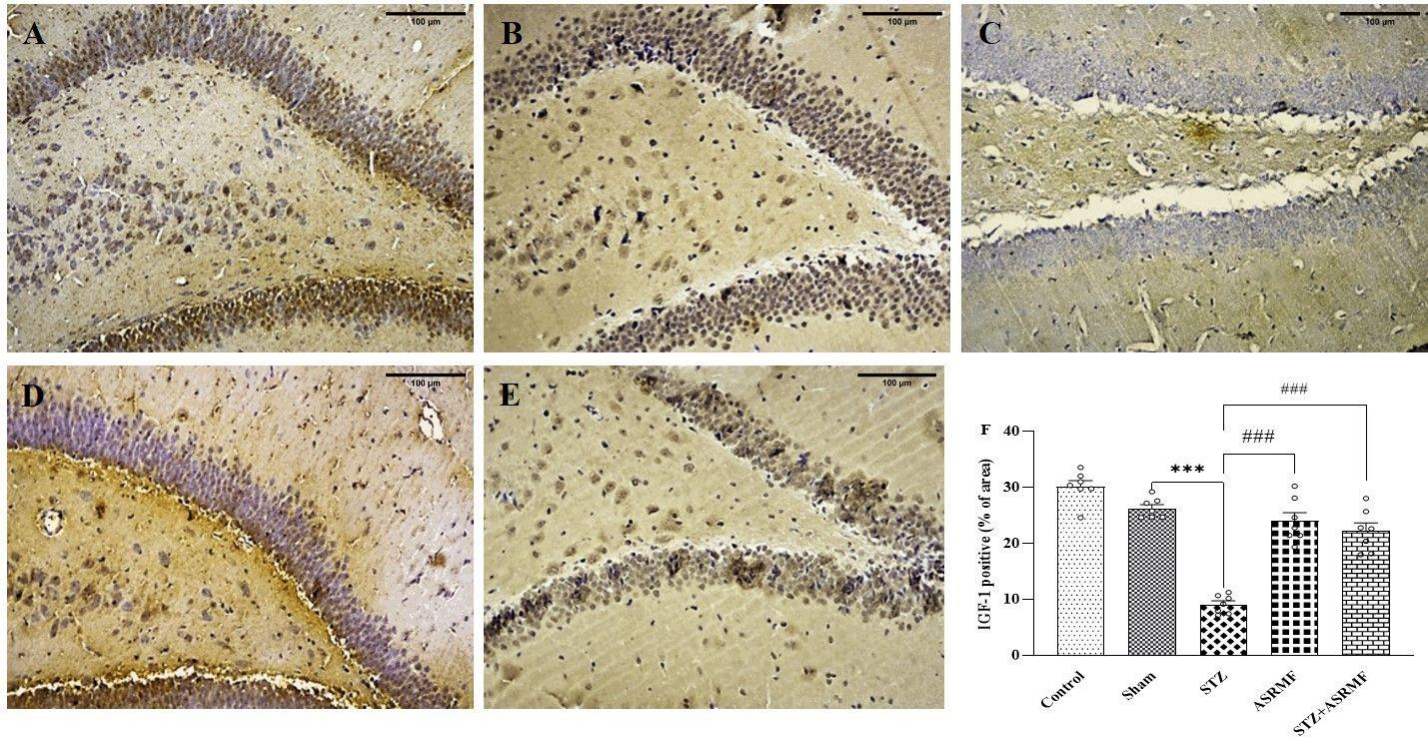
Illustrates the effect of ASRMF extract on the expression of the IGF-1 marker in the dentate gyrus (DG) region of the hippocampus. (A) The control group did not receive any intervention. (B) The sham received solvent intraperitoneally. (C) STZ groups was administered intraperitoneally injection of streptozotocin to induce diabetes. (D) Rats that received ASRMF extract by gavage for 15 days, (E) STZ-treated rats that received ASRMF extract by gavage for 15 days. (F) The quantification analysis of IGF-1 staining. Compared to the sham group, the STZ group exhibited a significant decrease in IGF-1 marker expression (***p<0.001). However, administration of the ASRMF extract on the STZ+ ASRMF extract group led to a marked improvement, with a substantial increase in IGF-1 levels compared to the untreated STZ group (###p<0.001). The data are presented as mean ± SEM, with n=7 animals per group. Scale bar: 100 μm, magnification 40X). Brown color indicates IGF-1 positivity.

IGF-1 expression in the dentate gyrus (DG) of the hippocampus varied significantly across groups (F (4, 30) = 51.6, p<0.001, Figure 4F). The STZ group exhibited a marked reduction in IGF-1 immunoreactivity compared to the sham group (9.09 ± 0.62 vs. 26.2 ± 0.67, p<0.001, Figure 4C). Administration of ASRMF extract significantly restored IGF-1 levels in the STZ+ASRMF extract group (22.2 ± 1.41, p<0.001, Figure 4E). There were no significant differences in IGF-1 expression between the sham and control groups.

### Hippocampal inflammatory cytokine expression 

**Figure 5:**
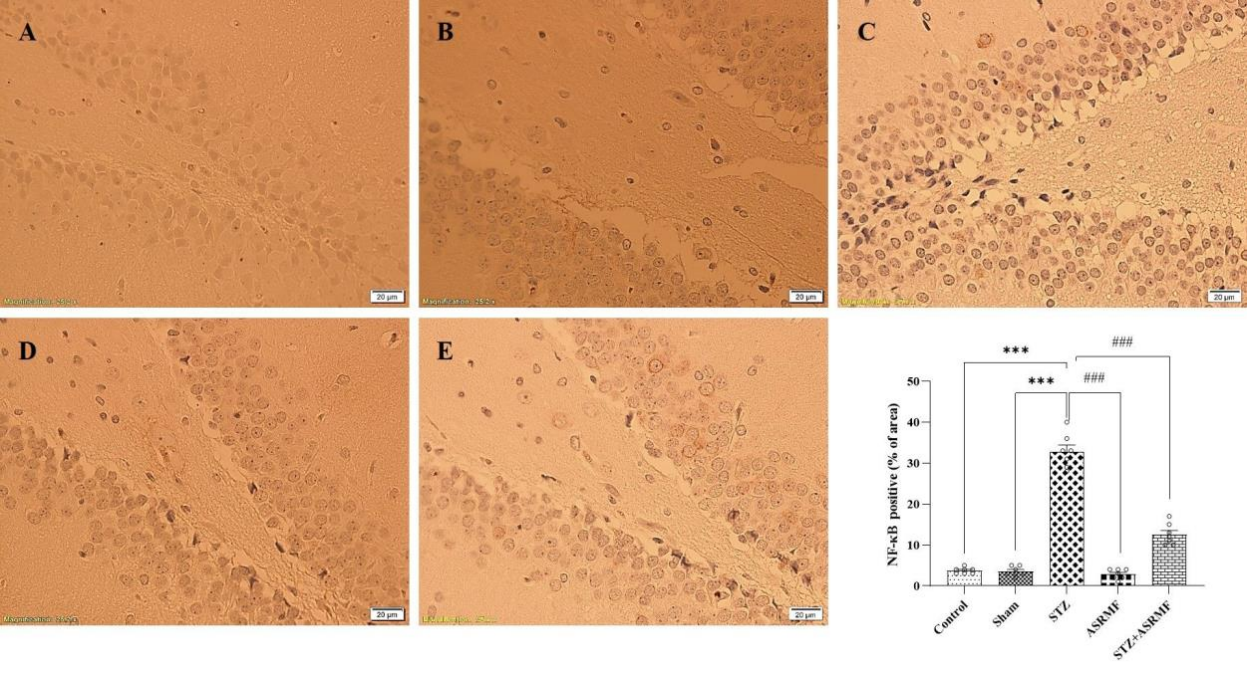
Immunohistochemistry analysis of NF-κB p65 phosphorylation in the hippocampal dentate gyrus (DG). (A) The control group did not receive any intervention. (B) The sham received solvent intraperitoneally. (C) STZ groups was administered intraperitoneally injection of streptozotocin to induce diabetes. (D) Rats that received ASRMF extract by gavage for 14 days, (E) STZ-treated rats that received ASRMF extract by gavage for 14 days. (F) The quantification analysis of NF-κB staining. Statistical analysis revealed significant differences, with the STZ group showing impaired performance compared to the sham group (***p<0.001), and the ASRMF extract treatment showing improvements in the STZ group (#p<0.05, ###p<0.001). The data are presented as mean ± SEM, with n=7 animals per group. (Scale bar: 20 μm, magnification 40X). Brown color indicates NF-κB positivity.

NF-κB levels were significantly elevated in the DG region of the hippocampus in the STZ group compared to the sham group (p<0.001). Treatment with ASRMF extract significantly reduced NF-κB levels in the STZ+ASRMF extract group (F (4, 30) = 209, p<0.001, Figure 5). Similar to IGF-1 expression, no significant differences were observed between the sham and control groups.

### Hippocampal Nissl staining

**Figure 6:**
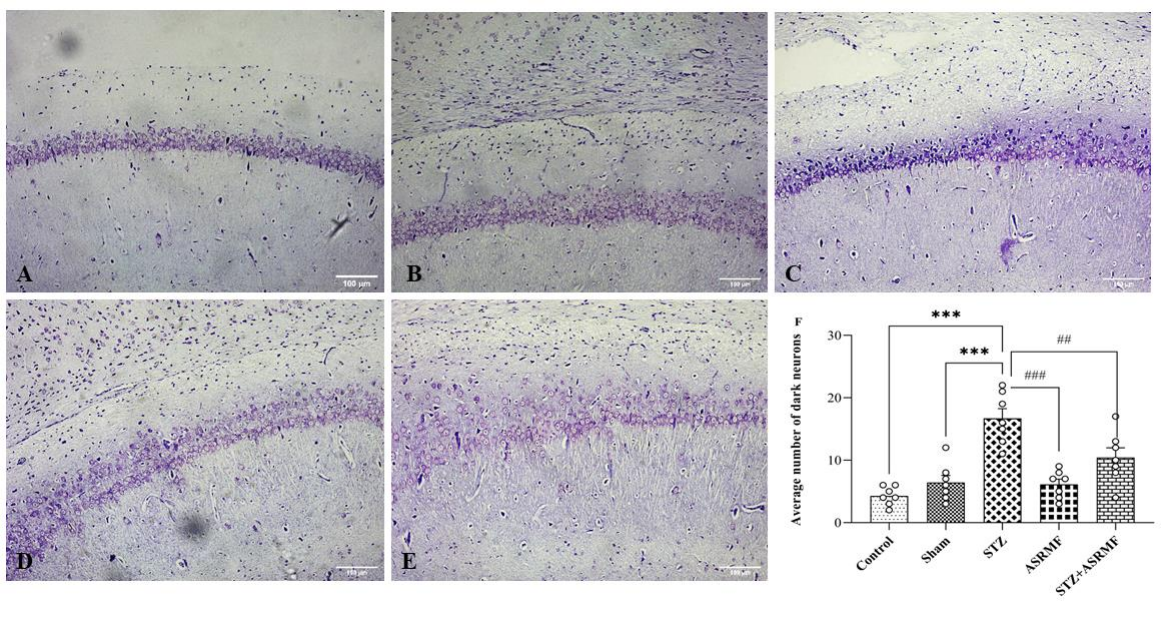
Photomicrographs of Cresyl violet (Nissl)-stained sections of the hippocampal CA1 region in different experimental groups and quantification of dark neurons. Representative images illustrate the hippocampal CA1 region across experimental groups: (A) Control, (B) Sham, (C) STZ, (D) ASRMF extract, and (E) STZ+ASRMF extract. The STZ group showed a pronounced increase in dark neurons, indicative of neuronal damage in the hippocampus. Notably, treatment with ASRMF extract in the STZ+ASRMF extract group (E) markedly reduced the number of dark neurons compared to the STZ group. Quantitative analysis of the average number of dark neurons per mm in the CA1 region (F). Data are presented as mean ± SEM (n=7). ***p<0.001 vs. Control and Sham; ##p<0.01 and ###p<0.001 vs. STZ. Scale bars: 100 μm, 200x magnification.

Quantitative analysis of Nissl-stained sections demonstrated no significant difference between the control, sham, and ASRMF extract groups but the STZ group exhibited a markedly higher number of dark neurons in the CA1 region of the hippocampus compared to sham group (F (4, 30) = 13, p<0.001, Figure 6). Notably, the STZ+ASRMF extract group displayed a significant reduction in the number of dark neurons relative to the STZ group, suggesting that ASRMF extract confers a neuroprotective effect against STZ-induced neuronal damage.

## Discussion

Our findings demonstrate that STZ-induced diabetes is associated with significant spatial memory impairment, corroborating prior research on its adverse effects on cognitive function. The observed reduction in hippocampal IGF-1 expression and the increase in NF-κB levels reinforce the role of neuroinflammation and disrupted neurogenesis in the pathogenesis of cognitive decline in diabetic models. This study is the first to reveal that ASRMF extract effectively ameliorates STZ-induced spatial memory impairment. The treatment not only increased hippocampal IGF-1 levels but also significantly decreased NF-κB expression, highlighting its dual neuroprotective and anti-inflammatory actions. Improved Morris water maze (MWM) performance in the STZ + ASRMF group emphasizes the extract ability to enhance learning and memory, possibly by mitigating the neuroinflammatory context.

Neuroinflammation, a key contributor to cognitive impairment, is characterized by elevated pro-inflammatory cytokines. Rats with diabetes-related cognitive decline exhibit a significant release of pro-inflammatory cytokines (Ahmad et al. 2022). In diabetes, inflammatory processes contribute to neuronal loss, synaptic dysfunction, and impaired insulin signaling, exacerbating memory deficits (Chen et al. 2022; Li and Hölscher 2007), increased Aβ production and tau hyperphosphorylation (Ortiz et al. 2022; Yuan and Wang 2017). 

STZ targets pancreatic β-cells, causing DNA damage and necrosis, which disrupts insulin production and glucose regulation (Szkudelski and medicine 2012). Insulin and its receptors are crucial for brain function, affecting cognition and synaptic plasticity, with dysfunction linked to cognitive decline (Yaribeygi et al. 2023).

In this study, we waited for four weeks after STZ administration before evaluating memory impairment to ensure the development of a stable and chronic diabetic state in the experimental animals.

Our finding that glucose levels remained elevated after 28 days suggests persistent hyperglycemia. Allowing several weeks for diabetes to progress ensures that hyperglycemia is persistent and severe, closely modeling chronic diabetes in humans and reducing the influence of residual endogenous insulin production or partial recovery of pancreatic function (Ahmed et al. 2019; Talbot et al. 2024). Although the 28-day waiting period before treatment may seem lengthy, multiple recent studies confirm that hyperglycemia induced by STZ in rats is stable and sustained throughout this timeframe. Studies demonstrate that STZ causes lasting pancreatic β-cell damage, resulting in persistent hyperglycemia and a disrupted but stable glucose regulatory system. Therefore, a 28-day post-STZ period is appropriate and necessary to establish a consistent diabetic state, ensuring reliable baseline conditions for evaluating therapeutic interventions (Ghasemi and Jeddi 2023; Rehman et al. 2023). This waiting period is also critical for the emergence of neurobiological changes associated with chronic hyperglycemia—such as increased inflammation, altered neurotransmitter levels, and disruptions in signaling pathways including AKT and CREB—which are implicated in diabetes-related cognitive deficits. By providing sufficient time for these processes to develop, the study design enables a more accurate assessment of the long-term effects of diabetes on memory function (Jin et al. 2022; Tian et al. 2016).

Our study indicates that high-dose STZ injection induces cognitive impairment, likely due to hyperglycemia-induced metabolic disturbances in the hippocampus. Previous studies indicated that memory impairment is associated with both acute and chronic high glucose levels. Chronic elevations—even at lower levels—can lead to hippocampal damage and memory deficits. Therefore, high glucose levels within these ranges can induce memory impairment through vascular, metabolic, and structural brain changes (Kassab et al. 2019; Xu et al. 2016; Zheng et al. 2017). The administration of ASRMF extract significantly decreased elevated blood glucose levels in the STZ +ASRMF group, potentially mitigating the risk and severity of memory impairment linked to hyperglycemia.

The CA1 region of the hippocampus is essential for memory formation and is particularly vulnerable to damage in models of diabetes and neurodegeneration studies using STZ-induced diabetic rats have shown that neuronal damage in the CA1 region correlates with impaired memory performance (Zhang et al. 2019; Zhao et al. 2016). In our study, STZ injection significantly increased the number of dark neurons and damaged the CA1 area, correlating with impaired memory performance. However, treatment with ASRMF extract not only improved memory but also reduced the number of damaged neurons in the CA1 region, as observed in Nissl-stained sections, indicating its neuroprotective effects on hippocampal structure.

STZ-induced spatial learning and memory dysfunction, as confirmed in our study, aligns with prior reports showing the role of inflammation in memory deficits and hippocampal neuron loss (Tamaddonfard et al. 2013; Wang et al. 2017). STZ +ASRMF group indicated significant improvement in MWM data. 

There is currently no direct evidence in the available scientific literature specifically demonstrating that *Allium saralicum *M. Fritsch extract crosses the blood-brain barrier (BBB). However, studies indicated that bioactive compounds such as allicin, carvacrol, and vitamin E, which are major constituents found in *Allium sativum*, have been shown to possess the ability to cross the blood-brain barrier (BBB). (Abbasloo et al. 2023; Hyun et al. 2013; Lee and Ulatowski 2019; Mohammadi et al. 2023a; Sánchez-Martínez et al. 2022). These findings suggest that the therapeutic effects of *Allium saralicum* on the brain may be attributed to the BBB permeability of its active constituents. 

The IGF-1/IGF1R signaling pathway essential in neurogenesis, synaptic plasticity, and neuronal survival. IGF-1, produced in the hippocampus, plays a critical role in supporting neural health by influencing dentate gyrus (DG) progenitor cells and activating the PI3K/Akt-CREB signaling pathway which promotes anti-apoptotic mechanisms and neuronal survival by inhibiting glycogen synthase kinase 3 (GSK3). Additionally, IGF-1 exhibits significant anti-inflammatory effects by regulating pro-inflammatory cytokine levels (Labandeira-Garcia et al. 2017; Witkowska-Sędek and Pyrżak 2020). Reduced IGF-1 levels in the STZ group correlate with impaired memory, consistent with literature linking IGF-1 deficits to age-related and diabetes-associated spatial learning and memory decline (Chattopadhyay and Shubayev 2009; Frater et al. 2018). IGF-1 exerts protective effects by activating PI3K/Akt pathways, suppressing pro-apoptotic signals like glycogen synthase kinase 3 (GSK3) (Loprinzi 2019). The restoration of IGF-1 levels by ASRMF treatment suggests its potential to enhance hippocampal neurogenesis and synaptic function, as evidenced by improved MWM performance. 

ASRMF impact on reducing NF-κB levels highlights its potential as an anti-inflammatory agent. NF-κB, a transcription factor involved in inflammation and oxidative stress, exacerbates cognitive decline in diabetes and neurodegenerative conditions (Ma and Long 2016). By modulating NF-κB activity, ASRMF may reduce the production of pro-inflammatory cytokines, improve insulin signaling, and enhance neuronal survival.

The antioxidant, anti-inflammatory, and neuroprotective properties of ASRMF make it a promising candidate for therapeutic development. Its ability to modulate IGF-1 and reduce NF-κB levels positions it as a potential intervention for diabetes-related cognitive decline and other inflammatory neurological disorders. 

Previous studies on ASRMF have established its safety and efficacy in reducing blood glucose levels and mitigating oxidative stress, further supporting its utility in managing diabetes and its complications (Mohammadi et al. 2023a). While the current findings illuminate key mechanisms underlying the neuroprotective effects of ASRMF, further research is needed to explore its impact on other signaling pathways involved in memory and inflammation, such as the AMPK and mTOR pathways. Additionally, investigations into the extract bioavailability, dosage optimization, and long-term effects are critical to advancing its therapeutic potential. 

ASRMF demonstrates significant neuroprotective effects in an STZ-induced model of spatial learning and memory impairment. By enhancing IGF-1 expression and reducing NF-κB levels, ASRMF alleviates neuroinflammation and improves memory function. These findings provide a foundation for further studies to explore its therapeutic potential for neurodegenerative and metabolic disorders.

## Data Availability

The raw data that support the findings of this study are available on request from the corresponding author's email address.
